# Detection and Attribution of Atmospheric Precipitable Water Changes since the 1970s over China

**DOI:** 10.1038/s41598-019-54185-z

**Published:** 2019-11-26

**Authors:** Jingpeng Zhang, Tianbao Zhao, Aiguo Dai, Wenyu Zhang

**Affiliations:** 1 College of Natural Resources and Environment, Northwest A&F University, Yangling, Shaanxi 712100 China; 2 College of Atmospheric Sciences, Key Laboratory for Semi-Arid Climate Change of the Ministry of Education, Lanzhou University, Lanzhou, Gansu 730000 China; 3 Key Laboratory of Regional Climate-Environment Research for East Asia, Institute of Atmospheric Physics (IAP), Chinese Academy of Sciences (CAS), Beijing, 100029 China; 4 Department of Atmospheric and Environmental Sciences, University at Albany, SUNY, Albany, NY 12222 USA; 5 School of Geoscience and Technology, Zhengzhou University, Zhengzhou, Henan 450001 China

**Keywords:** Attribution, Hydrology

## Abstract

Atmospheric water vapor increases as air temperature rises, which causes further warming. Thus, understanding the underlying causes of atmospheric water vapor change is vital in climate change research. Here, we conducted detection and attribution analyses of atmospheric precipitable water (PW) changes from 1973–2012 over China using an optimal fingerprinting method by comparing the homogenized radiosonde humidity data with CMIP5 model simulations. Results show that the increase in water vapor can be largely attributed to human activities. The effect of anthropogenic forcing (ANT) can be robustly detected and separated from the response to the natural external forcing (NAT) in the two-signal analysis. The moistening attributable to the ANT forcing explains most of the observed PW increase, while the NAT forcing leads to small moistening. GHGs are the primary moistening contributor responsible for the anthropogenic climate change, and the effect of GHGs can be also clearly detected and successfully attributed to the observed PW increases in a three-signal analysis. The scaling factor is used to adjust the CMIP5 model-projected PW changes over China and the observation-constrained future projections suggest that atmospheric water vapor may increase faster (slower) than that revealed by the raw simulations over whole (eastern) China.

## Introduction

As the most dominant greenhouse gas in the atmosphere^1–3^, water vapor plays a crucial role in the global energy and hydrological cycles and atmospheric circulation^4,5^. It affects our weather and climate^6–10^ as well as the biosphere^11^. According to the theoretical principles^12^, model simulations^2,13,14^ and observations^12,15–19^, atmospheric water vapor is expected to increase as air temperature rises, at a rate of ~7%/K approximately following the Clausius-Clapeyron equation for saturation vapor pressure^12^. However, how much or to what extent the increase in atmospheric water vapor can be attributed to anthropogenic influence and external natural forcing during the past several decades has not been well quantified so far.

Detection and attribution of climate change to quantify the relative contributions from human activities and natural forcing remain a scientific challenge^20,21^. Many previous studies on various climatic variables indicated that human activities are a major contributor to the recent climate change^22–24^. With respect to atmospheric water vapor, the last assessment report of the Intergovernmental Panel on Climate Change pointed out that an anthropogenic influence on increases in surface specific humidity has already been detected with medium confidence^25^. This is mainly based on Willett *et al*.^26^, who identified a significant increase in global surface specific humidity from 1973–1999 that is largely attributable to human influences. At the same time, Santer *et al*.^27^ analyzed lower tropospheric moisture content from 1988 to 2006 derived from satellite observations and found that the anthropogenic fingerprint in atmospheric water vapor simulated by a set of 22 different climate models was identifiable with high statistical confidence. Further study showed that the detection and attribution of anthropogenic influence on atmospheric water vapor is not sensitive to models used^28^.

Detection and attribution of regional climate changes are more challenging due to the lower signal-noise ratio that occurs at smaller spatial scales^14,20^. Over China, contributions of human activities to historical climate changes have been widely investigated in recent studies, most of them have focused mainly on temperature and precipitation^22–24,29–32^. Few studies have quantified anthropogenic impacts on recent water vapor changes. A lack of reliable humidity data has been a major inhibitor in the detection and attribution studies of atmospheric water vapor changes^20,25^. Dai *et al*.^33^ developed a new approach and applied to homogenize twice-daily radiosonde humidity data to produce a homogenized long-term data set. The homogenized humidity data performs well in exhibiting spatially coherent long-term trends, comparing more favorably with GPS PW than the raw radiosonde data^10,33^. This homogenized humidity data set has been used to quantify recent water vapor changes^3,33–35^.

In this article, we firstly utilized the multi-model simulations from the Phase 5 of the Coupled Model Intercomparison Project^36^ (CMIP5) together with the homogenized radiosonde humidity data to investigate the contributions by human activities and natural forcing to the observed recent changes in atmospheric precipitable water (PW) over China. Then, the detection and attribution results were applied to produce observation-constrained future projections that may be more realistic than the raw model output. See Materials and Methods section for detailed information about the datasets and methods.

## Observed and Model-Simulated Trends

**Figure 1:**
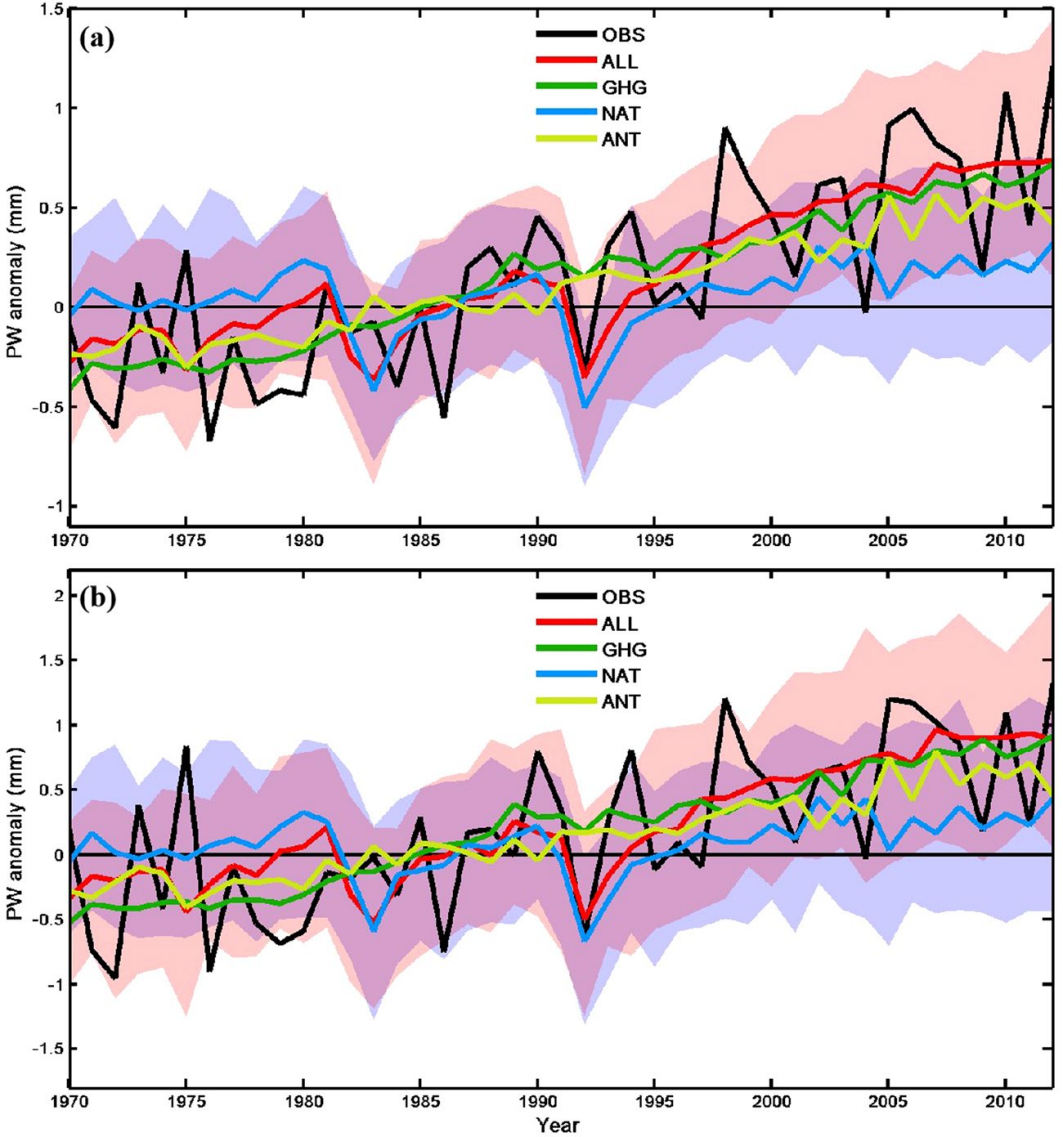
Annual mean PW anomalies for (**a**) whole China and (**b**) eastern China (east of 105°E) from observations (black line) and multi-model ensemble mean under ALL (red line), GHG (green line), NAT (blue line) and ANT (yellow-green line) forcing during 1970–2012. Pink and blue shadings show the 5–95% ranges of the individual model simulations from ALL and NAT experiments, respectively. The dark pink color indicates the overlapped area by the other two colors.

We first investigate the interannual variations and long-term changes of PW over China during 1970–2012. The PW variations over eastern China (east of 105°E) are highly consistent (correlation coefficient r = 0.96 and 0.99 that are statistically significant at the 5% level, respectively) with those over whole China in both observations and model simulations (Fig. 1). The observed mean PW (black line in Fig. 1) exhibits a clear upward trend, which is ~0.29 mm decade^−1^ over whole China and 0.32 mm decade^−1^ over eastern China (statistically significant at the 5% level). These are slightly higher than the PW trend (0.24 mm decade^−1^ is statistically significant at the 5% level) over 1973–2011 derived using global radiosonde data^35^. The PW increases are accelerated since the mid-1980s, with the upward trend of ~0.34 and 0.38 mm decade^−1^ from 1986–2012 for whole China and eastern China (statistically significant at the 5% level), respectively. It should be emphasized that the PW changes for the recent decades may partly result from internal decadal-multidecadal variability besides externally forced changes. On interannual time scales, the maximum value around 1998 results largely from the 1997/1998 El Niño event. The PW decrease during 1991–1992 is caused by the Pinatubo volcanic eruption in June 1991^4^.

The steady rise of PW in ALL experiments (red line in Fig. 1) agrees well with the observations, with an upward trend of 0.25 and 0.31 mm decade^−1^ over whole China and eastern China (statistically significant at the 5% level), respectively. The observed PW is well within the 5–95% ranges (pink shading in Fig. 1) of the individual ALL model simulations, which also reproduce the PW decrease after the Pinatubo volcanic eruption in 1991. Compared with the ALL case, the GHG-induced PW change (green line in Fig. 1) shows smaller variations with a nearly linear upward trend of ~0.27 and 0.34 mm decade^−1^ for whole China and eastern China (statistically significant at the 5% level), respectively. Similar to the GHG case, the ANT case also shows a steady upward trend of ~0.19 and 0.24 mm decade^−1^ for whole China and eastern China (statistically significant at the 5% level), respectively. In contrast, the NAT forcing (blue line in Fig. 1) contributes little to the PW change from 1970–2012, with a trend of 0.06 and 0.07 mm decade^−1^ for whole China and eastern China, respectively, except for the drop after the Pinatubo eruption in 1991. This result indicates that the NAT forcing cannot explain the PW increase over China, as the observed variations and changes are outside its 5–95% range (Fig. 1). Since atmospheric water vapor content depends strongly on air temperature, recent PW trends in observations and model simulations are consistent with temperature changes over China^3,32,34,37^.

**Figure 2:**
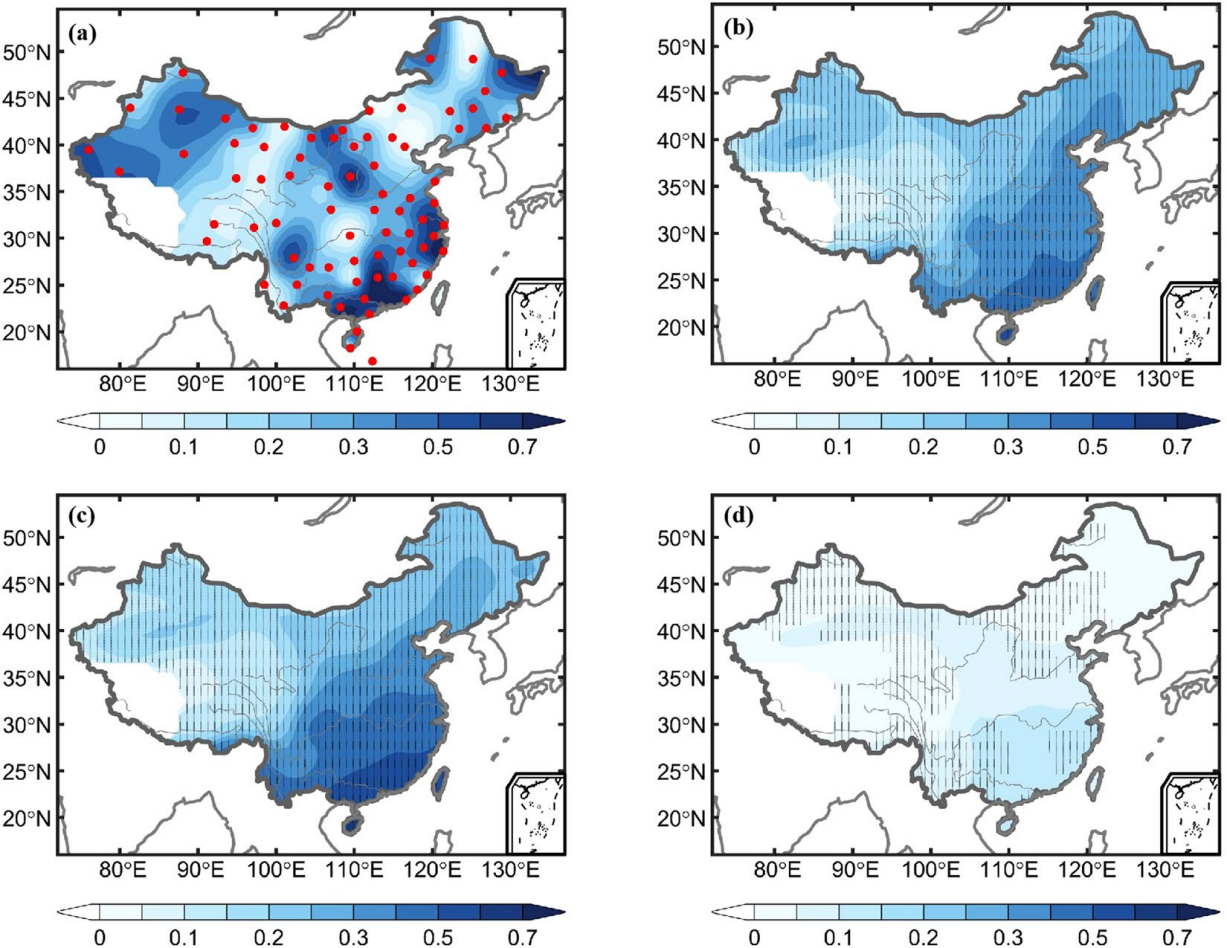
Annual trends (mm decade^−1^) of PW during 1970–2012 as computed from (**a**) observation and multimodel-simulated responses to (**b**) ALL, (**c**) GHG, and (**d**) NAT forcings. The red dots in (**a**) represent the 78 radiosonde stations used in this study, and the stippling in (**b–d**) indicates at least 80% of the individual simulations agree on the sign of the trend.

Figure 2 shows the spatial distributions of the PW trends over China from observations and model simulations under the ALL, GHG, and NAT forcings during 1970–2012. The observed PW trend patterns are consistent with earlier findings in Zhao *et al*.^34^. The ALL simulations capture the broad patterns of increasing trends seen in the observations, which may contain sampling noises and effects of internal variability that likely contributed to larger spatial variations in Fig. 2a. The PW trend patterns from ALL and GHG simulations are similar, with a maximum in Southeast China and decreasing towards the Northwest (Fig. 2b,c). However, the PW trend magnitude from GHG forcing is larger than that from the ALL case over southeastern China. Compared with ALL and GHG simulations, the NAT forcing results in little PW change over China, with a slight increase over southeastern China (Fig. 2d).

### Detection and attribution analysis

**Figure 3:**
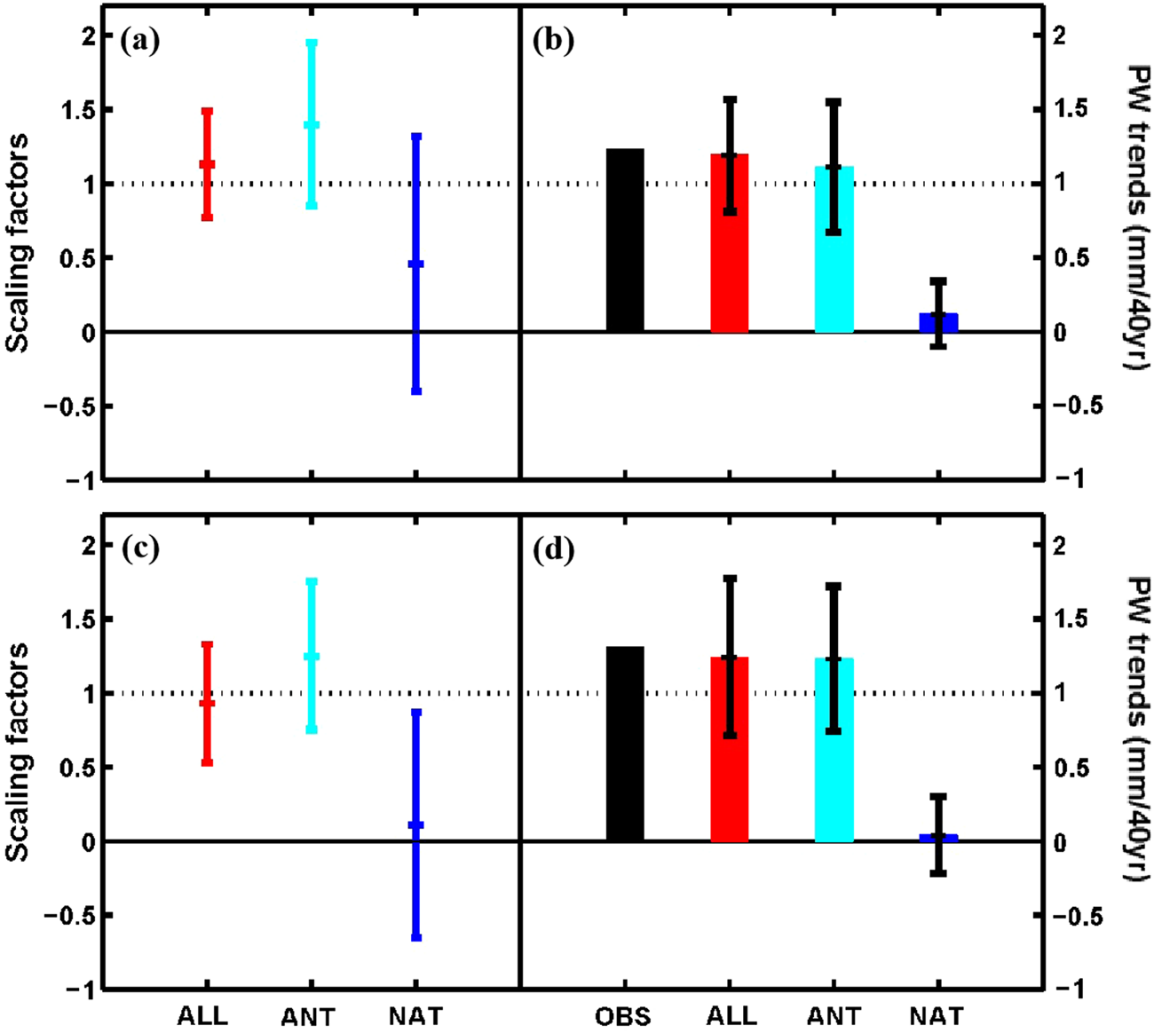
(left) Best estimates (the middle point) of the scaling factors using the regularized optimal fingerprint method and their 90% confidence intervals and (right) corresponding re-scaling trends and their 90% confidence intervals from a single-signal (ALL) analysis and a two-signal analysis (ANT and NAT) of 5-year mean PW during 1973–2012 over whole China (**a,b**) and eastern China (**c,d**). In the single-signal analysis, the observed PW is regressed onto the model-simulated response to ALL forcing. In the two-signal analysis, the observed PW is regressed using the total least squares algorithm onto the model-simulated responses to ANT and NAT simultaneously^41^. Trends for observations and model simulations are estimated based on linear least square regression.

Using the ROF approach, we performed detection and attribution analyses of the PW changes over China during 1973–2012 using ALL experiments in the single-signal analysis and ANT and NAT experiments in the two-signal analysis of the time series of non-overlapping 5-year means of PW anomalies over whole China and eastern China. The scaling factors and their 90% confidence intervals for the ALL case and the ANT and NAT case are shown in Fig. 3a,c and Table 1. The best estimate of the scaling factor for the ALL case from the single-signal analysis is 1.13 (90% confidence interval is 0.77~1.49) for whole China and 0.93 (0.53~1.33) for eastern China. These results indicate that the ALL forcing-induced PW trend is detectable in the PW over China, and that the observed and model-simulated changes are consistent with each other, as the 90% confidence interval contains 1.0 but not zero. For China as a whole, the best estimate of the scaling factor exceeds one, which indicates the model-simulated response underestimates the observed PW changes by about 12% [=100 × (1/β − 1)]. For eastern China, however, the model-simulated response overestimates the observed PW changes by about 7.5%.

**Table 1:** Scaling factors and PW trends estimated from single-signal analysis, two-signal analysis and three-signal analysis over China during 1973–2012.

Regions	Scaling factors						Trends (mm/40 yr)						
	single-signal	two-signal		three-signal			OBS	single-signal	two-signal		three-signal		
	ALL	ANT	NAT	GHG	ANTnoGHG	NAT		ALL	ANT	NAT	GHG	ANTnoGHG	NAT
Whole China	1.13 (0.77~1.49)	1.40 (0.85~1.95)	0.46 (−0.40~1.32)	1.52 (0.73~2.31)	0.42 (−0.60~1.44)	0.54 (−0.38~1.46)	1.23	1.19 (0.81~1.57)	1.11 (0.67~1.55)	0.12 (−0.10~0.34)	1.64 (0.79~2.49)	−0.12 (−0.41~0.17)	0.14 (−0.10~0.38)
Eastern China (east of 105°E)	0.93 (0.53~1.33)	1.25 (0.75~1.75)	0.11 (−0.65~0.87)	1.71 (0.80~2.62)	0.72 (−0.40~1.84)	0.09 (−0.81~0.99)	1.31	1.24 (0.71~1.77)	1.23 (0.74~1.72)	0.04 (−0.22~0.30)	2.39 (1.12~3.66)	−0.29 (−0.75~0.17)	0.03 (−0.27~0.33)

The PW trends are adjusted by the corresponding scaling factors, and the values in parentheses are their 90% confidence interval. All of the underlined values denote that the raw (unadjusted) trends both of observations and simulations are statistically significant at the 5% level.

From the two-signal analysis, it is clear that, the effect of the ANT forcing is clearly detectable (i.e., *β* exceeds zero), and the observed and ANT forcing-induced PW changes are consistent with each other (i.e., *β* includes one) for both whole and eastern China, although the best estimate of the scaling factor from ALL forcing is closer to the observed than that from ANT forcing (Fig. 3a,c). However, the effect of the NAT forcing is undetectable since the minimum *β* is less than zero, and the best estimate of *β* is close to zero over eastern China (Fig. 3c). This indicates that the observed PW changes over both whole and eastern China can be largely attributed to the ANT forcing, while the NAT forcing has little contribution, although their combination (i.e., ALL) produces a better match with the observed trends.

The contributions from ALL, ANT and NAT forcings to the observed PW trends, which are calculated using the “robust-fit method” that considers the effects of outliers and end points^38^, can be quantified by multiplying the model-simulated trend by the scaling factors and their 90% confidence intervals. These estimated PW trends attributable to the ALL, ANT and NAT forcings are shown in Fig. 3b,d and Table 1. Their best estimates for the ALL forcing case are 1.19 and 1.24 mm/40 yr over whole China and eastern China, respectively, which are slightly less than the trends from observations (which contain contributions from internal variability), which are 1.23 and 1.31 mm/40 yr. The ANT forcing explains most of the observed PW changes, accounting for 1.11 (0.67∼1.55) and 1.23 (0.74∼1.72) mm/40 yr over whole and eastern China, respectively; while the trends attributed to the NAT forcing are quite small, accounting for only 0.12 (−0.10∼0.34) and 0.04 (−0.22∼0.30) mm/40 yr for the two regions, respectively (Fig. 3b,d). Thus, we conclude that the long-term PW changes in China during 1973–2012 is mainly due to the contribution from anthropogenic forcing rather than natural forcing.

**Figure 4:**
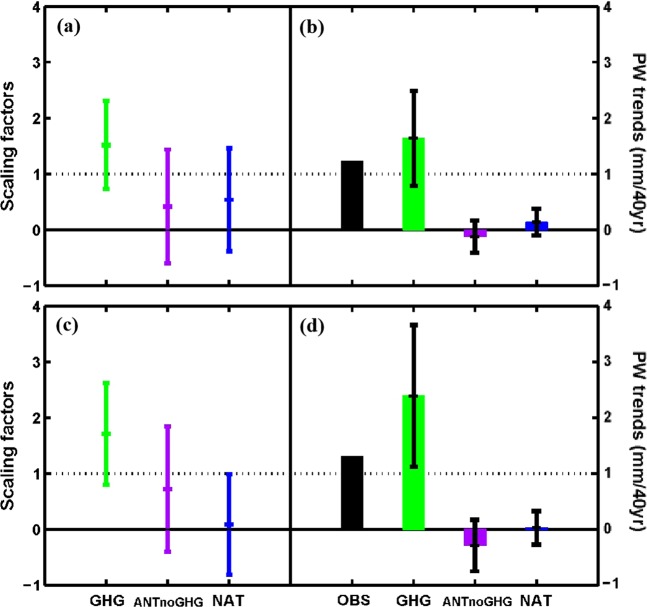
Same as Fig. 3, but for GHG, ANTnoGHG, and NAT signals in three-signal analysis.

To determine whether GHG is the most important factor among the anthropogenic forcings, we also conducted a three-signal detection analyses using GHG, ANTnoGHG, and NAT experiments. Figure 4a,c show that the GHG is not only clearly detected but also attributed successfully for China as a whole and its eastern region, and the magnitude of the scaling factor and its 90% confidence interval for GHG is larger than that for ANT in the two-signal analysis (Fig. 3a,c and Table 1). However, the effect of the other anthropogenic forcing (ANTnoGHG, 90% confidence interval of *β*: −0.60~1.44 and −0.40~1.84 for whole and eastern China) and NAT (90% confidence interval of *β*: −0.38~1.46 and −0.81~0.99) cannot be significantly detected. After re-scaling using *β*, the GHG forcing causes a PW increase of 1.64 (0.79~2.49) and 2.39 (1.12~3.66) mm/40 yr respectively for the two regions, whereas the PW trend induced by ANTnoGHG is mostly negative with −0.12 (−0.41~0.17) and −0.29 (−0.75~0.17) mm/40 yr for the two regions. Similar to the result of two-signal detection analysis, the trend attributable to the NAT forcing is relatively small (0.14 and 0.03 mm/40 yr for the two regions).

### Observation-constrained future projections

**Figure 5:**
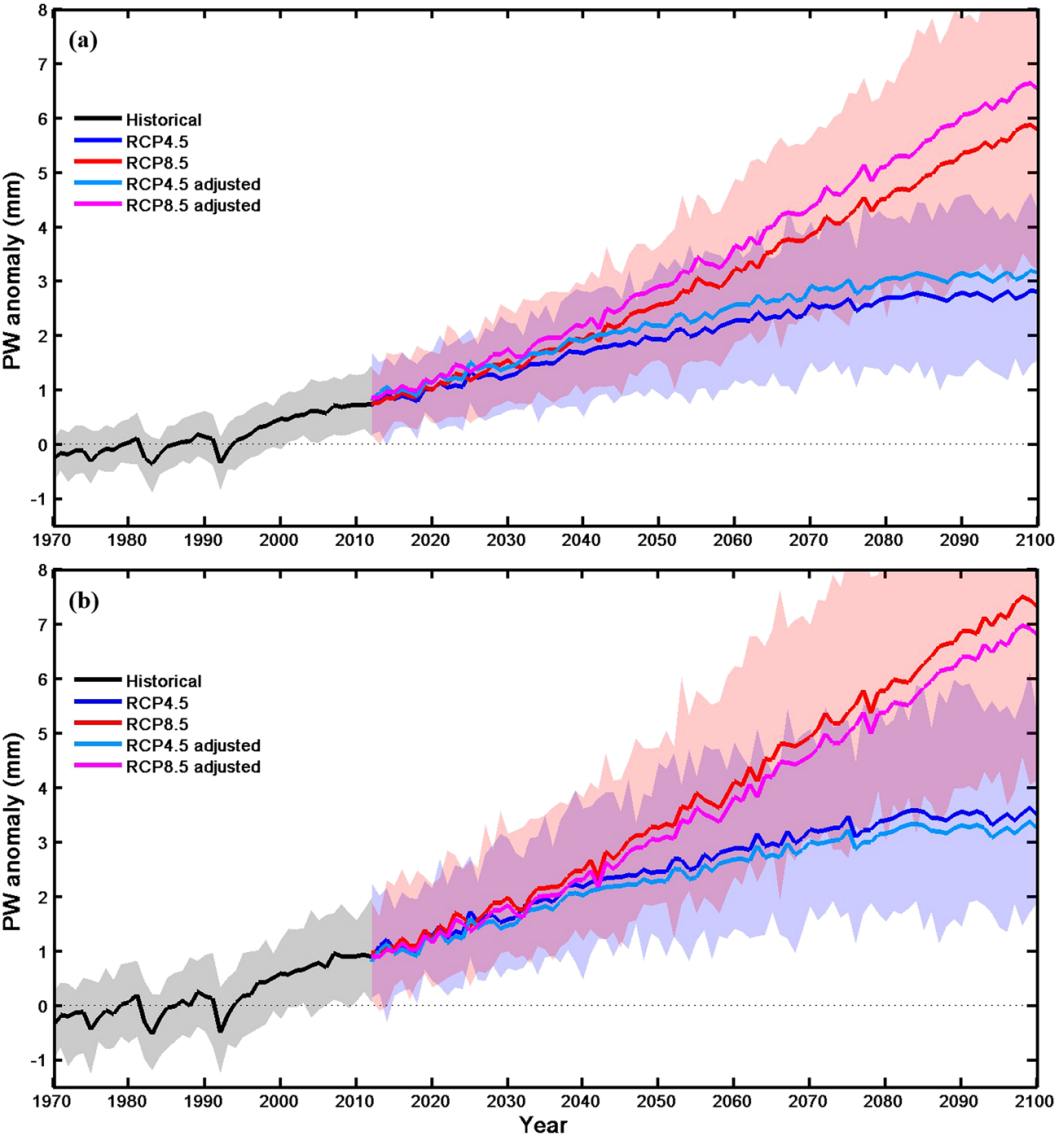
Time series of annual PW anomalies (relative to the 1970–1999 mean) over (**a**) whole China and (**b**) eastern China during 1970–2100. Future projections are based on the multi-model ensemble means under RCP4.5 (blue line) and RCP8.5 (red line). The light blue line and vivid red line represent the best estimates of the observation-constrained future PW projections under RCP4.5 and RCP8.5 scenarios, respectively. Pink and blue shadings show the spreads of 5–95% of the individual model simulations from RCP4.5 and RCP8.5 scenarios, respectively, while the dark pink color represents the overlapped areas of the two shadings.

From the single-signal detection based on ALL forcing, we found that the CMIP5 model-simulated PW underestimates for the PW change over whole China but overestimates it for eastern China. Thus, it is desirable to correct this systematic bias when using the CMIP5 model projections for future PW changes. To estimate future projections more accurately, we adjust the multi-model ensemble mean projection under the RCP4.5 and RCP8.5 scenarios for 2013–2100 by the best estimate of the scaling factors for the ALL forcing case; that is, the multi-model ensemble-mean projections are multiplied by the best estimate of the scaling factor. Figure 5 shows that the best estimates of the observation-constrained future PW projections are substantially higher than that projected by the raw simulations over whole China, but for eastern China, the results are just the opposite. For China as a whole, the best estimate of the PW increase in 2050 under the RCP4.5 and RCP8.5 are 2.17 mm (90% confidence interval: 1.48~2.86 mm) and 2.90 (1.98~3.82) mm, respectively, which are larger than the 1.92 mm and 2.57 mm in the raw simulations. For eastern China, the best estimate of the PW increase in 2050 under the RCP4.5 and RCP8.5 are 2.29 (1.31~3.27) mm and 3.03 (1.73~4.33) mm, respectively, which are less than the 2.46 mm and 3.26 mm in the raw simulations. The effect of the adjustment increases with time (Fig. 5).

## Summary and Discussion

Based on the CMIP5 multi-model simulations and homogenized radiosonde data, we analyzed the PW trends in China for the past few decades since the 1970s, and the relative contributions from external natural and anthropogenic forcings to the observed PW changes were also quantified. Then, we used the scaling factors from the detection and attribution analyses to produce observation-constrained future projections of PW change over China. The results showed that PW exhibits a clear increase, with an upward trend of 0.29 mm decade^−1^ for China as a whole and 0.32 mm decade^−1^ for eastern China during 1970–2012; and the trend has accelerated since the mid-1980s, with a positive trend of 0.34 mm decade^−1^ during 1986–2012 for China and a more rapid growth of 0.38 mm decade^−1^ for eastern China. The PW changes over China for the recent decades may be related to large internal climate variability and various external natural forcings, such as El Niño events and volcanic eruption.

The model-simulated PW changes under the ALL forcing can reproduce the increase pattern seen in the observations but cannot display the observed local characteristics. Compared with the ALL forcings, the GHG-induced PW changes lead to larger increases. By contrast, the NAT forcing contributes little to PW change from 1970 to 2012, although they do capture the effect associated with large volcanic eruptions. This finding indicates that the NAT forcing cannot explain the recent PW increase over China.

Utilizing the ROF approach, we performed detection and attribution analyses on the PW changes from 1973–2012 using ALL experiments for single-signal analysis and ANT and NAT experiments for two-signal analysis. The best estimate of the scaling factor β for ALL forcing in the single-signal analysis is 1.13 and 0.93 over whole China and eastern China, respectively, which indicates the model-simulated response underestimates (overestimates) the observed PW change from 1973–2012 for whole (eastern) China by about 12% (7.5%). In the two-signal analysis, the 90% confidence interval of the scaling factor β for ANT not only exceeds zero but also includes one, while it includes zero for NAT; which indicate that the observed PW increase over China can be mainly attributable to the ANT forcing, which can be separated from the NAT signal. After re-scaling PW trends using the scaling factors, the ANT forcing explains most of the observed PW changes, with the best estimate around 1.11 and 1.23 mm/40 yr from 1973–2012 over whole China and its eastern region. In contrast, the PW trends attributed to the NAT forcing are quite small, only around 0.12 and 0.04 mm/40 yr for the two regions.

In the three-signal detection analysis, the GHG forcing is clearly detected and attributed successfully (90% confidence interval of *β*: 0.73~2.31 and 0.80~2.62 for whole and eastern China), with a PW trend induced by GHG of 0.79~2.49 and 1.12~3.66 mm/40 yr for whole China and eastern China, respectively. This means that the GHG forcing is the most important factor among the anthropogenic forcings for the PW change over China during 1973–2012.

Based on the best estimate of scaling factor *β* for the ALL forcing case in the single-signal analysis, we calculated the observation-constrained future projections for PW over China. The adjusted future projections show substantially larger increases in atmospheric water vapor than that suggested by the raw simulations over whole China, but slower increases eastern China.

It should be recognized that our results likely contain uncertainties associated with model deficiencies in simulating climate response to a given external forcing, as well as uncertainties existed in the estimated historical forcings used by the CMIP5 model simulations. Furthermore, observational data over western China are sparse, especially for atmospheric humidity derived from radiosonde records^33^. Even for surface air temperature and precipitation, twentieth-century global trends estimated from different datasets can differ noticeably^39,40^. Thus, observational uncertainties may exist in our estimated PW changes, and hamper the detection and attribution results. In addition, the uncertainties in cloud microphysics and convective parameterizations applied in each climate model are considered as a major source for model errors and uncertainties in the accuracies of simulations of the PW. Further investigations are clearly needed into the uncertainties in the influence of anthropogenic forcings on climate variability.

## Materials and Methods

### Homogenized radiosonde humidity data

We used the homogenized twice-daily radiosonde humidity data from Dai *et al*.^33^. The homogenized humidity data are available for around 100 stations within China with over 80% of the time with observations during 1970–2012 (data after 2012 require new homogenization and thus are not used here). We selected 78 of these Chinese stations that had at least 90% of the time with observations during 1970–2012. The spatial distribution of the 78 stations is shown in Fig. 2a. Visual inspection suggests that these radiosonde stations are roughly uniformly distributed over most of China except the western region. Besides examining trend maps, we also analyzed the PW time series averaged over whole China and eastern China (east of 105°E), which has better station coverage (Fig. 2a) and is influenced heavily by the East Asian monsoon. Here, we focus on the changes and variations in column-integrated PW, which is concentrated mainly in the lower troposphere. The PW is calculated by integrating the specific humidity from the surface to 300 hPa using $PW=\frac{0.1}{g}{\int }_{300}^{{p}_{s}}qdp$, where PW is in mm, *g* (=9.8 m s^−2^) is the acceleration of gravity, *p*_*s*_ is surface pressure in hPa, *q* is specific humidity in g kg^−1^, and *p* is air pressure in hPa.

### CMIP5 Model simulations

CMIP5 model simulations were used to estimate the PW response to external forcings and the spread caused by internal climate variability. Here we utilized 68 historical simulations from 22 climate models to represent the response to all external forcings (ALL), 44 simulations from 10 models under greenhouse gas forcing (GHG) only, and 56 simulations from 11 models under natural forcing (NAT) only. The low-moderate representative concentration pathway (RCP4.5) simulations for 2006–2012 were used to extend the ALL forcing simulations as the historical simulations ended in 2005, while the GHG and NAT runs ended in 2012. We estimated the response to anthropogenic forcing (ANT) by differencing ALL and NAT cases, i.e., our ANT = ALL − NAT. Further, we derived another ANT case without the GHG effect (ANTnoGHG) by differencing ANT and GHG: ANTnoGHG = ANT − GHG. Additionally, we used pre-industrial control (CTL) simulations from 22 models to estimate the internal climate variability. These CTL simulations were divided into 289 segments of 40 years (the length of 1973–2012). Furthermore, the detection and attribution results were applied to constrain 21^st^ century projections under the RCP4.5 and RCP8.5 (a high emissions) scenarios from 22 models. The CMIP5 model simulations used here are listed in Table 2, more details can be seen at the CMIP5 website: http://cmippcmdi.llnl.gov/cmip5/.

**Table 2:** List of multi-model simulations used in this study.

Model Name	ALL	GHG	NAT	CTL	RCP
ACCESS1-0	1			12	1
ACCESS1-3	1	3	3	12	1
BCC-CSM1-1	1	1	1		1
CanESM2	5	5	5	24	1
CCSM4	6			12	1
CNRM-CM5		6	6	21	1
CSIRO-MR-3-6-0	10	10	10	12	
GFDL-CM3	3			12	1
GFDL-ESM2G	1			12	
GFDL-ESM2M	1			12	
GISS-E2-H	10	5	10	13	1
GISS-E2-H-CC	1			6	1
GISS-E2-R	10	5	10	13	1
GISS-E2-R-CC	1			6	1
HadGEM2-CC					1
HadGEM2-ES	4	4	4	14	1
INM-CM4					1
IPSL-CM5A-LR	4	4	3	25	1
IPSL-CM5A-MR	1		3	7	1
IPSL-CM5B-LR					1
MIROC5					1
MIROC-ESM	1			15	1
MIROC-ESM-CHEM	1			6	1
MPI-ESM-MR	3			25	
MRI-CGCM3	1			12	1
NorESM1-M	1	1	1	12	1
NorESM1-ME	1			6	
SUM(models)	68(22)	44(10)	56(11)	289(22)	22(22)

Numbers represent the ensemble sizes for the ALL, GHG, and NAT forcing and RCP scenarios. For the CTL simulations, numbers represent the number of 40-year chunks.

### Detection method

The optimal fingerprint method, which has been extensively used in detection and attribution studies^9,23,37,41–43^, is applied here to quantify the relative contributions from each individual external forcing. As an extension of the optimal fingerprinting method, the regularized optimal fingerprint (ROF) provides a more objective and accurate implementation of detection and attribution with a specific estimate of the covariance matrix^44^. This method assumes that the climate response signal and the internal noise are linearly additive^25^; that is, the observed changes are the sum of externally-forced change and internally-generated variations. We first conducted a single-signal analysis, with the observed PW variations regressed onto the model-simulated response to ALL forcing for whole China and eastern China (thus, no spatial patterns were used in the analysis). The ALL-forcing signal is estimated using the multi-model ensemble mean, as the uncorrelated internal variations are largely smoothed out in the ensemble averaging. We then performed a two-signal analysis by regressing the observed spatio-temporal variations onto the response patterns from the ANT and NAT simulations, in which ANT = ALL − NAT, to examine the relative contributions of anthropogenic and natural forcing to the observed PW changes. Finally, we also conducted a three-signal analysis using GHG, NAT and ANTnoGHG (i.e. ANT-GHG) signals to estimate the relative contributions of the individual external forcings. In these analyses, the observed regional PW series is regressed against the model-simulated PW series:

1 $$P{W}_{OBS}={\beta }_{ALL}P{W}_{ALL}+\varepsilon $$

2 $$P{W}_{OBS}={\beta }_{ANT}P{W}_{ANT}+{\beta }_{NAT}P{W}_{NAT}+\varepsilon $$

3 $$P{W}_{ALL}=P{W}_{ANT}+P{W}_{NAT}$$

4 $$P{W}_{OBS}={\beta }_{GHG}P{W}_{GHG}+{\beta }_{ANTnoGHG}P{W}_{ANTnoGHG}+{\beta }_{NAT}P{W}_{NAT}+\varepsilon $$

5 $$P{W}_{ALL}=P{W}_{GHG}+P{W}_{ANTnoGHG}+P{W}_{NAT}$$

where *PW*_*OBS*_ is the regionally-averaged PW (see section 2.3 for more details) from observations; *PW*_*ALL*_, *PW*_*ANT*_, *PW*_*NAT*_, *PW*_*GHG*_ and *PW*_*ANTnoGHG*_ denote the regional PW response to ALL, ANT, NAT, GHG and ANTnoGHG forcing, respectively; *β*_*ALL*_, *β*_*ANT*_, *β*_*NAT*_, *β*_*GHG*_ and *β*_*ANTnoGHG*_ are the unknown regression coefficients or scaling factors, and *ε* represents regression residual term. In detection and attribution analysis, the observational data series may not be independent and identically distributed (i.i.d). For such cases, the covariance structure of the observation data is needed in the linear regression, and CTL run data are often used to estimate such covariance matrix^41^. Here we used half of the CTL data segments for estimating the covariance in order to derive the *β* factor. The other half was used to estimate its confidence interval. Equations (1, 2 and 4) represent the single-, two- and three-signal attribution analysis, respectively. It should be noted that *β*_*NAT*_ estimated in the two-signal analysis (Eq. 2) and three-signal analysis (Eq. 4) may be different. To account for the sampling error in the model-simulated externally-forced PW signals as they are estimated from a limited number of ensemble runs, the total least squares algorithm^41^, which accounts for uncertainties in both variables of the regression, was used to estimate the scaling factor *β* in these equations.

The above method provides a technique to determine whether the signal from the anthropogenic and natural forcings could be detected and whether the influence of the anthropogenic forcing, especially greenhouse gas increases, could be separated from the naturally-forced signal and the internal variability. We used the residual consistency test proposed by Allen and Tett^45^ to evaluate whether the model-simulated variability is small enough. If the 90% confidence internal of the scaling factor differs from zero, it means this forcing factor is successfully detected. And if the detected factor’s 90% confidence internal includes unity and above zero, then the observed change is consistent with the simulated response to this external forcing. The deviation of *β* from unity is often used to infer the mean bias in the models in simulating the forced signal, under the assumption that internal variations in observations do not contribute to the estimated forced component (the right-hand terms in Eqs. 1, 2 and 4 except *ε*). If the best estimate of *β* is above (below) unity, then the models underestimate (overestimate) the forced signal in observations^22,32^.

### Data processing

The forced response or signal is represented using the multi-model ensemble mean, which is an equally weighted arithmetic average of the ensemble mean of the simulations from individual models. To facilitate the analysis and comparison, PW anomalies (relative to the 1970–1999 climatology) from observations and model simulations were re-mapped onto a common 1° lat × 1° lon grid. The Cressman interpolation technique^46^ with a maximum influence radius of 1000 km was used in the gridding of the station PW anomalies, while bi-linear interpolation was used to remap the model data. In the detection and attribution analysis, for each grid box we converted the annual PW data into a series of non-overlapping five-year means over 1973–2012 (i.e., for 1973–1977, 1978–1982, …, 2008–2012) to shorten the time dimension and to reduce the variability in the observations and noise in the climate signals. To further shrink the noise, following Wen *et al*.^22^, the 5-year mean PW data were projected onto empirical orthogonal functions (EOFs). We included only those leading EOFs in our subsequent detection and attribution analysis that together explained about 80% of the total variance. The grid boxes without observations were masked out in analyzing the model data, so that the spatial sampling is the same for the observations and model data. Weighted by cosine of the latitude at the center of each grid box, we constructed the regional averages over whole China and eastern China using the EOF-reconstructed data to increase the signal-to-noise ratio^47^, and these regional PW data series were used in Eqs. (1–5). To estimate the noise from natural variability and for statistical testing of the estimated scaling factor, we derived 289 segments of non-overlapping 40-year chunks from the CTL simulations by 22 models. We similarly averaged the 40-year data series into 5-year means and split the 289 chunks into two halves, with one used for optimization (i.e., for estimating the noise term ε in Eqs. (1, 2 and 4), and the other for statistical testing of the estimated slopes in these equations^22^.
